# NecroX-5 Can Suppress Melanoma Metastasis by Reducing the Expression of Rho-Family GTPases

**DOI:** 10.3390/jcm10132790

**Published:** 2021-06-25

**Authors:** Gue-Tae Moon, Ji-Hyun Lee, Sang-Hyun Jeong, Song-Wan Jin, Young-Min Park

**Affiliations:** 1 Department of Biomedicine & Health Sciences, The Catholic University of Korea, #222 Banpo-daero, Seocho-gu, Seoul 06591, Korea; therlone@naver.com (G.-T.M.); yiji1@hanmail.net (J.-H.L.); 2 Department of Dermatology, Seoul St. Mary’s Hospital, College of Medicine, The Catholic University of Korea, #222 Banpo-daero, Seocho-gu, Seoul 06591, Korea; 3 Department of Advanced Convergence Technology, Korea Polytechnic University, #237 Sangidaehak-ro, Siheung-si 15073, Gyeonggi-do, Korea; jsh12589@naver.com; 4 Department of Mechanical Engineering, Korea Polytechnic University, #237 Sangidaehak-ro, Siheung-si 15073, Gyeonggi-do, Korea

**Keywords:** NecroX-5, melanoma, metastasis, Rho-family GTPase, Cdc42, Rac1, RhoA

## Abstract

NecroX-5 (NX-5) is a cell-permeable necrosis inhibitor with cytoprotective effects. Although it has been reported to inhibit lung and breast cancer metastasis by modulating migration, its therapeutic effect on melanoma metastasis is still unknown. In this study, we examined the anti-metastatic effect of NX-5 on melanoma cell lines and its related therapeutic mechanism. The anti-metastatic effect of NX-5 on melanoma cell lines was determined using a transwell migration assay. We performed a quantitative real-time polymerase chain reaction and western blot analysis to measure changes in the expression of mRNA and protein, respectively, for major mediators of Rho-family GTPases after NX-5 treatment in melanoma cells. In addition, after constructing the 3D melanoma model, the expression of Rho-family GTPases was measured by immunohistochemistry. NX-5 (10 μM and 20 μM) treatment significantly reduced melanoma cell migration (*p* < 0.01). Additionally, NX-5 (20 μM) treatment significantly decreased the mRNA and protein expression levels of Cdc42, Rac1, and RhoA in melanoma cells compared with the untreated group (*p* < 0.001 and *p* < 0.05, respectively). Immunohistochemistry for our 3D melanoma model showed that Cdc42, Rac1, and RhoA were constitutively expressed in the nuclei of melanoma cells of the untreated group, and NX-5 treatment decreased their expression. These results demonstrate that NX-5 can suppress melanoma metastasis by reducing the expression of Rho-family GTPases.

## 1. Introduction

Melanoma is a fatal disease that accounts for 90% of skin cancer-related deaths, and metastasis easily occurs with a poor prognosis [1,2]. Most melanomas metastasize to distant organs such as the liver, bones, and brain; skin, subcutaneous tissue, and lymph nodes are the primary sites of metastasis [3]. Although several anti-cancer drugs and immunotherapeutic agents have been tried, thus far, their therapeutic efficacy against invasion and metastasis is still limited [4,5].

Cancer metastasis is a process of cell migration, epithelial-mesenchymal transition (EMT), intravascular invasion, the potential circulation of tumor cells, and deposition to secondary organs. In cancer cells, EMT increases mobility and invasiveness and gives cancer cells the ability to degrade the extracellular matrix (ECM) [6,7].

Rho-family GTPases have Cdc42, Rac1, and RhoA as the main subfamily and play important roles in various cell functions including angiogenesis and invasion, cytoskeletal structure formation, cell proliferation, apoptosis, and cancer metastasis [8,9,10]. In melanoma, Cdc42 contributes both amoeboid and mesenchymal movement and overall tumor cell invasion. [11]. Rac1 is known to enhance the elongated protruding movement of melanoma cells by promoting nuclear alterations through PAK1 and the tubulin cytoskeleton [12]. Additionally, RhoA is activated by the chemokine CXCL12, which triggers a cellular polar response, leading to rounded, amoeboid movement [13,14]. When Rho-family GTPases move to the cell membrane and are activated, they induce cancer cell transplantation and metastasis through downstream target molecules [15]. Mediation of Rho-protein activity can advance tumor cell metastasis by disrupting epithelial-sheet organization, increasing cell motility, and promoting ECM degradation. Rho-family GTPase overexpression is associated with the progression of some cancers, including breast and lung cancers [16].

NecroX-5 (NX-5) is a derivative of the NecroX series of compounds and has been introduced as a drug for inhibiting cancer metastasis [17]. NX-5 has been reported to reduce the calcium concentration in cells, thereby reducing the invasion ability of breast cancer cells [18]. However, the anti-metastatic effect of NX-5 on melanomas has not yet been fully elucidated. In this study, we examined whether NX-5 has the potential to inhibit the metastatic response of melanoma cells.

## 2. Materials and Methods

### 2.1. Cell Culture and Reagents

Human melanoma cell lines (A375P, A375SM) were obtained from the Korean Cell Line Bank (KCLB, Seoul, Korea). The A375P cell was maintained in DMEM supplemented with 10% (*v*/*v*) fetal bovine serum (FBS), 1% (*v*/*v*) *L*-glutamine and penicillin–streptomycin. A375SM was maintained in MEM supplemented with 10% (*v*/*v*) FBS and 1% (*v*/*v*) *L*-glutamine and penicillin–streptomycin. All cells were cultured in a 37 °C, 5% CO_2_ humidified incubator. NecroX-5 (Enzo Life Sciences, Farmingdale, NY, USA), ZCL278, NSC23766, and CCG-1423 (Selleck Chemicals, Houston, TX, USA) were dissolved in dimethyl sulfoxide (DMSO) for each condition and dose. 3-(4,5-Dimethylthiazol-2-yl)-2,5-diphenyltetrazolium bromide (MTT) and DMSO were purchased from Sigma–Aldrich (St. Louis, MO, USA).

### 2.2. Cell Viability Assay

Melanoma cells (1 × 10^4^ /mL) were seeded onto 96-well plates with 100 μL/well culture medium. After 24 h, the medium was replaced with serum-free medium, then treated with NX-5 (1–50 μM) for 24 h, when cell viability was examined using MTT. MTT reagent (20 μL, 5 mg/mL PBS) was added to each well. The cells were further incubated at 37 °C for 4 h. After careful removal of the medium, 100 μL of buffered DMSO was added to each well, and the plates were shaken. Cellular metabolism was determined by recording the absorbance of samples at 540 nm in a VersaMax Microplate Reader (Molecular Devices, Sunnyvale, CA, USA). The effect of each compound on cell growth was assessed as a percentage of cell viability where vehicle-treated cells were taken as 100% viable.

### 2.3. Cell Migration Assay

Melanoma cell migration was evaluated using 24-well Transwell plates (Costar, Corning Inc., Corning, NY, USA). The appropriate number of A375P and A375SM cells were added to the upper chamber of transwell plates with 8μm pores with or without NecroX-5 at 10 μM or 20 μM and incubated for 24 h. The upper surface of the membrane was wiped with a cotton-tipped applicator to remove residual cells. Cells in the bottom compartment were fixed and stained with hematoxylin, and a photographic image of the bottom of the transwell membrane was taken. Migration of the human melanoma cell lines was determined by counting three fields randomly selected at ×100–200 magnification from the upper chamber to the bottom side of the membrane.

### 2.4. Quantitative Real-Time Polymerase Chain Reaction (RT-qPCR)

Melanoma cells were seeded at a density of 1 × 10^4^ mL/well in plates. After being cultured for 24 h, the medium was replaced with serum-free medium. After starvation, the cells were treated with NX-5 (10, 20 μM), ZCL278 (10 μM), NCS23766 (10 μM), or CCG-1423 (100 nM). The negative control cells were incubated with sterile distilled water. Total RNA from tissues was isolated with TRIzol reagent (Invitrogen, Carlsbad, CA, USA) according to the manufacturer’s protocol and reverse-transcribed to cDNA using a cDNA synthesis kit (Doctor Protein, Seoul, Korea). RT-qPCR was performed using a CFX96™ Real-Time PCR Detection System^®^ (Bio-Rad, Hercules, CA, USA) with MG 2X qPCR MasterMix Ⅱ (SYBR Green) (MGmed, Seoul, Korea) and specific primers. Conditions of the RT-qPCR reaction consisted of an initialization step for 10 s at 95 °C followed by two-step PCR for 45 cycles of 95 °C for 5 s (denaturation) and 58–60 °C for 30 s (annealing/extension). Results were normalized to the level of glyceraldehyde 3-phosphate dehydrogenase (GAPDH) gene expression. The analysis of relative gene expression data was conducted using the 2^−ΔΔCT^ method.

### 2.5. Immunohistochemistry

All specimens were embedded in optimal cutting temperature compound (Sakura Tissue-Tek Xpress, Torrance, CA, USA). Immunohistochemistry was performed on 5 µm thick sections obtained from 4% paraformaldehyde-fixed, paraffin wax-embedded tissue. Following dewaxing and rehydration steps, antigen retrieval was performed using citrate buffer. Sections were blocked with peroxidase blocking solution (Dako, Santa Clara, CA, USA) for 15 min. The sections were incubated with Cdc42 antibody (1:1000, Abcam), Rac1 antibody (1:1000, Invitrogen), and RhoA antibody (1:2000, Novus Biologicals) overnight at 4 °C. After rinsing with phosphate-buffered saline (PBS), the sections were incubated with Dako REAL™ EnVision/HRP (DAKO, Carpinteria, CA, USA) at room temperature for 2 h, and were visualized with substrate-chromogen solution. Counterstaining was performed with Mayer’s hematoxylin (Dako). Stained tissue samples were observed using a Leica light microscope DMI 5000B (Leica, Wetzlar, Germany).

### 2.6. Western Blot

Whole-cell lysates were prepared in radio-immunoprecipitation assay (RIPA) buffer (Thermo Fisher Scientific, Waltham, MA, USA) on ice with an added phosphatase inhibitor cocktail (PhosSTOP, Roche, Basel, Switzerland). The protein concentrations for each sample were determined using the bicinchoninic acid (BCA) protein assay kit (ThermoFisher Scientific) according to the manufacturer’s directions. Equal amounts of protein (20 μg) were resolved by electrophoresis on 15% sodium dodecyl sulphate (SDS) polyacrylamide gels and transferred to nitrocellulose membranes. The membranes were blocked with 5% bovine serum albumin (BSA) in Tris-buffered saline (TBS-T) and incubated at room temperature for 2 h with primary antibodies against Cdc42 (1:1000, ab64533, Abcam, Cambridge, MA, USA), Rac1 (1:1000, PA1-091, Invitrogen, Carlsbad, CA, USA), and RhoA (1:2000, NB100-91273, Novus Biologicals, Littleton, CO, USA). These membranes were washed out in TBS-T and incubated with secondary antibodies diluted 1:5000 for 1 h (IgG HRP-linked, anti-rabbit, and anti-mouse antibodies, respectively; Cell Signaling Technology, Danvers, MA, USA). The results were visualized using enhanced chemiluminescent (ECL) detection reagents (Millipore, Darmstadt, Germany).

### 2.7. Fabrication of Nanofibrous Membrane

Electrospinning was used to fabricate a nanofibrous membrane [19,20,21]. Briefly, the polycaprolactone (PCL) with a number-average molecular weight of 80,000 (440,744, Sigma-Aldrich, St Louis, MO, USA) was dissolved in a 9:1 mixture of chloroform (C0584, 99.5%, Samchun Pure Chemical Co., Ltd., Seoul, Korea) and dimethylformamide (Sigma-Aldrich) at a concentration of 17% by weight. During electrospinning, the temperature and relative humidity were 20–21 °C and 50–55%, respectively. The flow rate of the solution, voltage, and tip-to-collector-distance were maintained at 0.2 mL/h, 18.0–18.1 kV, and 18 cm. A rotating drum collector was used to collect continuously electrospun nanofibers. The rotating speed (5 rpm) of the collector drum was set low enough to prevent fiber alignment. The fabricated nanofibrous membrane was 35 ± 5 µm-thick. To evaporate the remaining chloroform, the membrane was placed in a fume hood at room temperature for 24 h. Then the nanofibrous membrane was soaked in 70% ethanol overnight for sterilization under an ultraviolet lamp. Finally, the nanofibrous membrane was washed twice using PBS (pH 7.4, Gibco, Rockville, MD, USA).

### 2.8. Printing Melanoma Cells and Aggregation

A375P cells were printed in a drop-on-demand manner on the nanofibrous membrane using a micro dispensing system (CT-M3-04 Micro Dispensing, Microfab, Inc., Plano, TX, USA). The cell suspensions in culture media (1 × 10^6^ cells/mL) were loaded in a 3 mL syringe. The syringe is assembled with a piezoelectric printer head and three-dimensional stage. The printer head has an inner diameter of 120 μm (MJ-ABL-01-120-8MX, Microfab, Inc.). Approximately 900 microdroplets, generated by the piezoelectric print-head, were dropped on the surface of the nanofibrous membrane. The printing frequency was set at 3 Hz, and the distance between the end tip of the printer head and the nanofibrous membrane surface is approximately 2 mm. The dropped cells formed an aggregation on the surface of the membrane because of highly porous and randomly intertwined nanofiber structures [19]. Immediately after the printing, the printed cell aggregates on the nanofibrous membrane were cultured with DMEM in 5% CO_2_ at 37 °C for 1 day to ensure stable formation of cell aggregates (Figure 4).

### 2.9. Development of Epidermis

The melanoma printed nanofibrous membrane was coated with collagen (2.5 mg/mL, collagen type I, Thermo Fisher Scientific) to enhance normal human epidermal keratinocytes (NHEK, Lonza Walkersville, MD, USA) adhesion, and the NHEK cells (2 × 10^5^ cell/cm^2^) were seeded with keratinocytes basal medium (KBM, Lonza) on the melanoma printed side of the membrane and incubated for 2 h in 5% CO_2_ at 37 °C for initial cell adhesion. After that, they were air-liquid interface (ALI) cultured in 6-well plates with keratinocytes differentiation medium: a 1:1 mixture of DMEM and KBM containing calcium chloride (Sigma-Aldrich) at 1.8 mM (Figure 4) for more than 7 days. The medium was changed every two or three days.

### 2.10. Statistical Analysis

All data are representative data from at least three independent experiments. Statistical analysis was performed via one-way analysis of variance (ANOVA) followed by Tukey’s multiple comparison test using GraphPad Prism, version 5.02, GraphPad Software (La Jolla, CA, USA). Unpaired t-tests were used for comparisons of the two groups. All data are expressed as the mean ± standard error of the mean (SEM). All graphs were generated using GraphPad Prism 5 (La Jolla, CA, USA). Statistical significance was considered when a *p*-value was less than 0.05 (* *p* < 0.05; ** *p* < 0.01; and *** *p* < 0.001).

## 3. Results

### 3.1. Viability of Human Melanoma Cells after Treatment with NecroX-5

**Figure 1:**
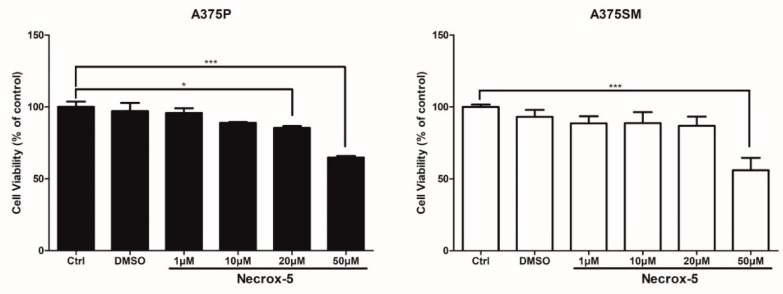
The effect of NecroX-5 treatment on cell viability in A375P and A375SM melanoma cells. Cells were treated with various concentrations of NecroX-5 for 24 h. Cell viability was measured using the MTT assay. Results are expressed as the mean ± SEM from the three independent experiments. * *p* < 0.05, *** *p* < 0.001.

A375P showed a significant decrease in cell viability at concentrations of 50 μM of NX-5 (35.7%, *p* < 0.001), whereas A375SM showed little to no change in cell viability at all concentration levels, with the exception of a significant decrease at the 50 μM concentration (37.4%, *p* < 0.001). Based on these results, the NX-5 concentrations of 10 and 20 μM with relatively high cell viability of A375P and A375SM were chosen for further study (Figure 1).

### 3.2. NecroX-5 Treatment Decreased Melanoma Cell Migration

**Figure 2:**
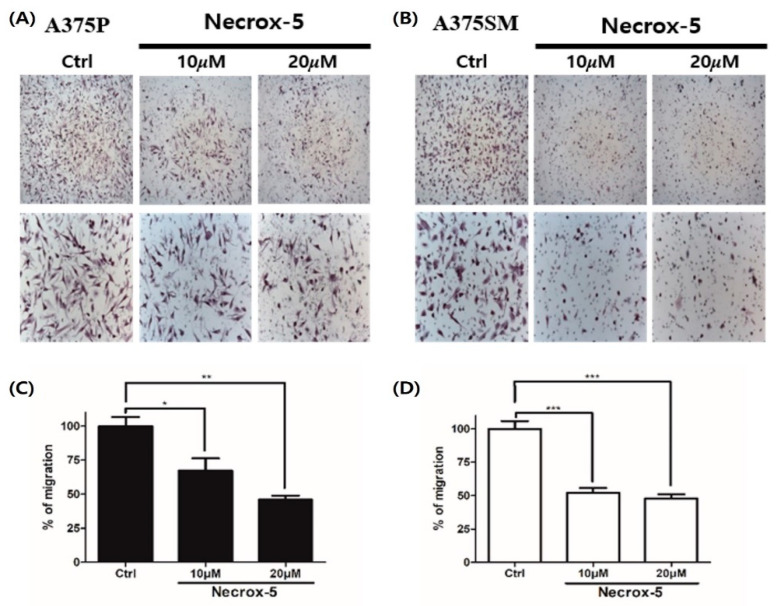
The effect of NecroX-5 treatment on melanoma cell migration. A375P and A375SM were inoculated in the upper chambers of transwell plates with 8-μm pores with or without NecroX-5 at 10 μM or 20 μM. (**A**,**B**) After 24 h, the bottom side of the membrane was stained with hematoxylin, and a photographic image of the bottom of the transwell membrane was taken. (**C**,**D**) Migration of human melanoma cell lines was determined by counting three fields randomly selected at ×100–200 magnification from the upper chamber to the bottom side of the membrane. Results are expressed as the mean ± SEM from the three independent experiments. * *p* < 0.05, ** *p* < 0.01, *** *p* < 0.001.

A375P cell migration was significantly decreased at 10 μM (32.7%, *p* < 0.05) and 20 μM (54%, *p* < 0.01) NX-5 concentrations. In addition, A375SM cell migration was also significantly decreased following treatment with 10 μM (47.8%, *p* < 0.001) and 20 μM (52%, *p* < 0.001) NX-5 concentrations (Figure 2). These results demonstrated that NX-5 can reduce melanoma cell migration.

### 3.3. NecroX-5 Treatment Reduced Melanoma Cell Migration by Reducing the Expression of Rho-Family GTPases

**Figure 3:**
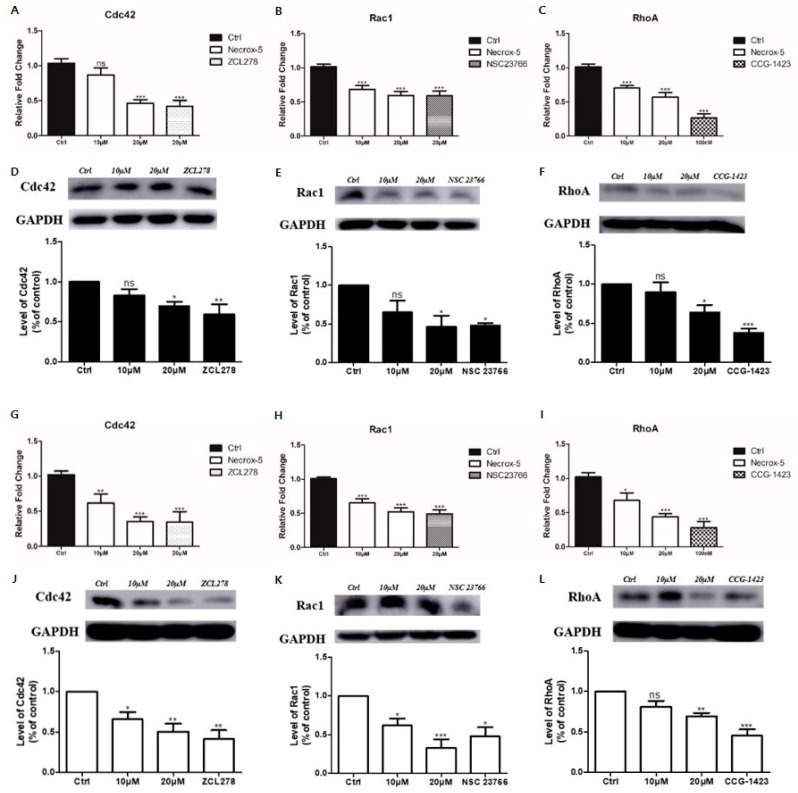
The effect of NecroX-5 treatment on the expression of Rho family GTPases (Cdc42, Rac1, and RhoA). A375P (**A**–**F**) and A375SM (**G**–**L**) were treated with 10 μM or 20 μM NecroX-5 for 24 h. The mRNA expression levels of Cdc42, Rac1, and RhoA were measured by qRT-PCR (**A**–**C**,**G**–**I**). The protein levels of Cdc42, Rac1, and RhoA were measured using western blot analysis (**D**–**F**,**J**–**L**). GAPDH was used as an internal control for mRNA and protein normalization (ZCL278 (10 μM), Cdc42 GTPase inhibitor; NCS23766 (10 μM), Rac GTPase inhibitor; CCG-1423 (100 nM), RhoA GTPase inhibitor). Results are expressed as the mean ± SEM from three independent experiments. * *p* < 0.05, ** *p* < 0.01, *** *p* < 0.001.

NX-5 (20 μM) treatment significantly decreased the mRNA expression of Cdc42, Rac1, and RhoA in A375 and A375SM cells (*p* < 0.001). However, unlike in A375SM cells, there was no significant difference in the mRNA expression of Cdc24 after NX-5 (10 μM) treatment in A375 cells. NX-5 (20 μM) treatment significantly reduced the mRNA expression of Cdc42, Rac1, and RhoA similar to Cdc42 inhibitor (ZCL278), Rac1 inhibitor (NSC23766), and RhoA inhibitor (CCG-1423) in A375P and A375SM (*p* < 0.001 for each) (Figure 3A–C,G–I). NX-5 (10 μM) treatment did not decrease the expression of Cdc42, Rac1, and RhoA proteins in A375 cells. NX-5 (20 μM) treatment significantly decreased the expression of Cdc42, Rac1, and RhoA proteins in A375P cells (*p* < 0.05) (Figure 3D–F). In addition, NX-5 (10 μM) treatment did not decrease the expression of RhoA proteins in A375SM cells (Figure 3L). NX-5 (20 μM) treatment significantly decreased the expression of Cdc42 (*p* < 0.01), Rac1 (*p* < 0.001), and RhoA (*p* < 0.01) proteins in A375SM cells (Figure 3J–L). These results indicate that NX-5 can inhibit melanoma cell migration by reducing the expression of Rho-family GTPases such as Cdc42, Rac1, and RhoA, which differs depending on NX-5 concentration and the melanoma cell line.

### 3.4. Necrox-5 Treatment Down-Regulated the Expression of Rho-Family GTPase in 3D Melanoma Model

**Figure 4:**
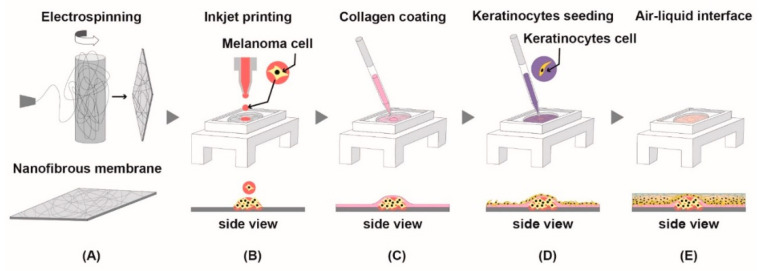
The process of making a 3D melanoma model. (**A**) Fabricating a nanofibrous membrane using electrospinning. (**B**) Printing and forming cell aggregation of human melanoma cells (A375P) using a micro dispensing system. (**C**) Coating the nanofibrous membrane with collagen. (**D**) Seeding the normal human epidermal keratinocytes on the membrane. (**E**) Air-liquid interface culture.

**Figure 5:**
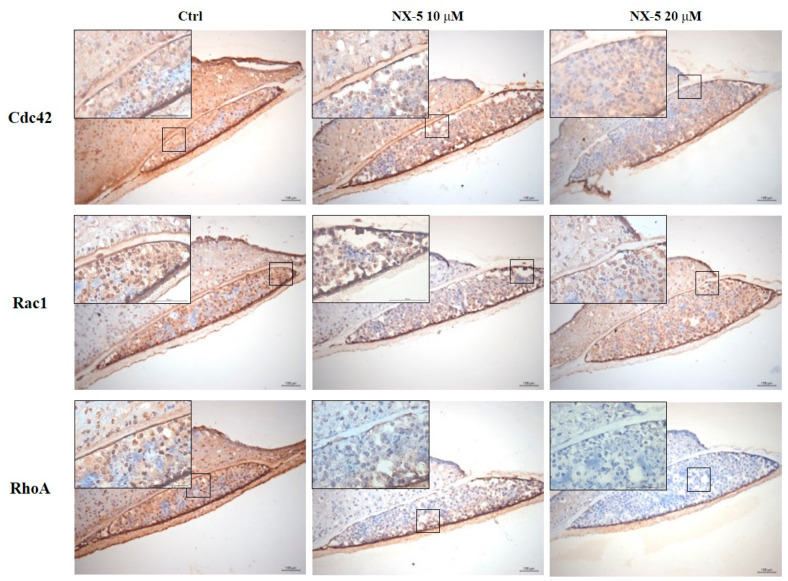
Immunostaining of the 3D melanoma model treated with NecroX-5. The models were treated with 10 μM or 20 μM NecroX-5 for 1 day. Immunostaining for expression of Cdc42, Rac1, and RhoA was performed (scale bars = 100–400 µm, boxed areas = 100 µm).

To confirm the anti-metastatic effect of NX-5 on melanoma cells, we constructed a 3D melanoma model (Figure 4A–E) and then measured the expression of Rho-family GTPases after NX-5 (20 μM) treatment (Figure 5). Immunohistochemistry showed that Cdc42, Rac1, and RhoA were constitutively expressed in the nuclei of melanoma cells of the untreated group, and NX-5 treatment decreased their expression. These results demonstrated that NX-5 treatment can down-regulate the expression of Rho-family GTPases in a 3D melanoma model.

## 4. Discussion

In this study, NX-5 treatment decreased A375P and A375SM cell migration. This result was elucidated by reducing the expression of Rho-family GTPases (Cdc42/Rac1/RhoA) in human melanoma cell lines after NX-5 treatment. NX-5 treatment has been shown to down-regulate the expression of Rho-family GTPases in the 3D melanoma model as well.

NX-5 is one of the derivatives of the NecroX series of compounds that effectively inhibit mitochondrial ROS generation and oxidize low-density lipoprotein cholesterol generation [22,23]. Nam et al. reported that regulation of intracellular ROS through NX-5 attenuated *p*-glycoprotein expression via inhibiting STAT1 phosphorylation [24]. Park et al. showed that NX-5 prevents metastasis by inhibiting the migration of human cancer cells including breast and lung cancers. They also found that NX-5 inhibits metastasis-related cell migration by down-regulation of AKT through inhibiting Ca^2+^ influx [18]. In this study, NX-5 treatment significantly reduced melanoma cell migration in the highly metastatic A375SM cell line as well as in the low metastatic A375P cell line.

In cancer, metastasis occurs when tumor cells migrate from their original site and invade other tissues in the 3D environment. There are two types of movement in tumor cell migration, mesenchymal and amoeboid modes, and both are involved in melanoma metastasis [25]. The Rho-family GTPases Cdc42, Rac1, and RhoA are key substances involved in the mesenchymal movement as well as amoeboid movement including actin dynamics and cell movements [26,27,28]. In addition, they are linked with MAPK and PI3K pathways involved in melanoma progression [16]. Overexpression of P29S mutation after UV exposure is reportedly involved in cancer proliferation and migration through Cdc42 and Rac1 [29,30]. RhoA has also been found to be involved in cell mobility in B16F10 melanoma cells [31]. ZCL278, a known Cdc42 inhibitor, directly blocks guanine nucleotide exchange factor (GEF) binding, and NSC23766, a Rac1 inhibitor, suppresses Rac-GTP loading by blocking GEF-mediated activation [32,33]. CCG-1423, a RhoA inhibitor, reduces RhoA-mediated transcription [34]. Our current study demonstrates that NX-5 significantly down-regulates mRNA and protein expression of Rho-family GTPases, which are important for metastasis in a 3D environment. In addition, NX-5 has the potential to have similar inhibitory effects as Cdc42 inhibitor, Rac1 inhibitor, and RhoA inhibitor in human melanoma cell lines. Taken together, our results suggest that NX-5 can replace known Rho-family GTPase inhibitors.

One of the strengths of this study is that a new melanoma model using 3D printing was applied. A 3D model of melanoma better represents the tumor environment found in patients than classical cell cultures. Especially, it is well known that the three-dimensional microenvironment influences cell functionality, phenotype, and morphology. We successfully demonstrate the fabrication of a 3D melanoma model using a drop-on-demand inkjet-based 3D printer and nanofibrous membrane which is advantageous in terms of maintaining the shape of cell aggregates after printing. Moreover, we make our model more clinically relevant by forming an epidermis layer on the top of the melanoma aggregates. The model described herein is highly reproducible and can be formed in a shorter period of time than existing 3D culture melanoma models [35] making it easier to access the experiment. Using the 3D melanoma model prepared in this way, the reduction in the expression of Rho-family GTPase (Cdc42/Rac1/RhoA) protein by NX-5 treatment was confirmed by immunohistochemical staining. We believe that this model could be used for further drug research on melanoma.

In conclusion, in this study, we showed that NX-5 treatment on human melanoma cell lines inhibited cell migration by suppressing the activity of Rho-family GTPases. This suggests the potential efficacy of NX-5 as a treatment modality for melanoma metastasis.

## Data Availability

The data presented in this study are available in the article.

## Abbreviations

ECM	Extracellular matrix
EMT	Epithelial-mesenchymal transition
NX-5	NecroX-5
